# Prevalence and determinants of anaemia among women of reproductive age in Aspirational Districts of India: an analysis of NFHS 4 and NFHS 5 data

**DOI:** 10.1186/s12889-024-17789-3

**Published:** 2024-02-12

**Authors:** Subhojit Let, Seema Tiwari, Aditya Singh, Mahashweta Chakrabarty

**Affiliations:** 1 Department of Geography, Banaras Hindu University, Varanasi, India; 2 Geography Section, Mahila Maha Vidyalaya, Banaras Hindu University, Varanasi, Uttar Pradesh India; 3 Girl Innovation, Research, and Learning (GIRL) Center, Population Council, New York, NY United States of America

**Keywords:** Anaemia, NFHS, Women's Health, Women of Reproductive age, Aspirational Districts, WRA, India, Hemoglobin

## Abstract

**Background:**

Over one-third of women worldwide suffer from anaemia. The prevalence of anaemia is particularly pronounced among women of reproductive age (WRA) in developing countries, such as India. No prior study has ever exclusively studied the prevalence of anaemia across the Aspirational Districts of India. Therefore, the purpose of this study was to examine the prevalence of anaemia across Aspirational Districts of India and to identify the determinants of anaemia among WRA in these districts.

**Methods:**

From the National Family Health Survey (NFHS)-4 (2015-16) and NFHS-5 (2019-21), data on 114,444 and 108,782 women aged 15–49 from Aspirational Districts were analyzed in our study, respectively. Bivariate statistics and multivariable binary logistic regression were used to identify the determinants of anaemia.

**Results:**

The national prevalence of anaemia among WRA has increased from 53% in NFHS-4 to 57% in NFHS-5 whereas anaemia among WRA in Aspirational Districts has increased from 58.7% in NFHS-4 to 61.1% in NFHS-5. Between 2015 and 2021, over 60% of Aspirational Districts experienced an increase in the prevalence of anaemia and one-fourth, specifically 29 out of 112, observed a rise by at least 10 percentage points (pp). Notably, there are significant variations in anaemia prevalence among districts, with Simdega and Udalgiri having the highest anaemia prevalence in NFHS-4 and NFHS-5 at 78.2% and 81.5%, respectively. During this period, Barpeta followed by Udalgiri of Assam have witnessed the maximum increase with 29.4% and 26.7% respectively. Moreover, pooled regression results show women with three to four children [AOR: 1.13, 95% CI: 1.08–1.17], women who breastfeed [AOR: 1.17, 95% CI: 1.13–1.20], Scheduled Tribe women [AOR: 1.39, 95% CI: 1.35–1.44], poorest women [AOR: 1.27, 95% CI: 1.22–1.33] and women those who consume fish occasionally [AOR: 1.14, 95% CI: 1.12–1.17] were more likely to be anaemic.

**Conclusion:**

The significant increase in anaemia among WRA in Aspirational Districts of India is a matter of concern. Given the rise in anaemia among WRA, determinants-based and district-specific measures must be designed and implemented to reduce the prevalence of anaemia among Aspirational Districts of India.

**Supplementary Information:**

The online version contains supplementary material available at 10.1186/s12889-024-17789-3.

## Background

Anaemia is a condition where the count and size of red blood cells, or the concentration of hemoglobin, drop below a defined threshold [1]. According to World Health Organization (WHO), pregnant women are considered anaemic if their hemoglobin concentration is less than 11.0 g/dl, while non-pregnant women are considered anaemic if their hemoglobin concentration is below 12.0 g/dl [2]. Anaemia has three main causes: nutritional deficiencies, infectious diseases, and genetic disorders related to hemoglobin [3, 4]. Iron-deficiency anaemia stands out as the most prevalent type in developing nations [5, 6]. A global analysis indicates that anaemia affects 27% of the world’s population, with iron deficiency being the primary cause [7, 8]. Inadequate intake of essential nutrients such as iron, vitamin B12, and folic acid, coupled with excessive consumption of tea, coffee, and certain spices, can lead to nutritional deficiencies that often result in anaemia [3, 9, 10]. Similarly, genetic disorders, such as vitamin A, riboflavin, and folate deficiencies, sickle cell disease, glucose-6-phosphate dehydrogenase deficiency, as well as conditions like malaria, human immunodeficiency viruses (HIV), and tuberculosis, significantly contribute to the development of anaemia [3, 11]. It is a significant public health concern impacting approximately half a billion women aged 15–49 years [12]. In 2019, WHO estimates 30% (539 million) of non-pregnant women and 37% (32 million) of pregnant women aged 15–49 years were affected by anaemia [12].

Women of reproductive age (WRA) are one of the most vulnerable groups to this condition, caused by poor dietary intake of essential micronutrients, chronic diseases, heavy menstruation, infections, genetic disorders, and reproductive-related blood loss [1, 12–15]. Anaemia among WRA associated to several adverse health outcomes, such as poor pregnancy outcomes, preterm birth, stillbirth, increased susceptibility, low birth weight, loss of productivity, fatigue, breathlessness, dizziness, maternal morbidity, and mortality [16–18]. Moreover, anaemia can negatively impact infants’ and children’s cognitive and physical development [19–22].

Anaemia holds crucial importance in the context of achieving Sustainable Development Goals (SDGs), particularly SDG-2 (Zero Hunger) and SDG-3 (Good Health and well-being) [23, 24]. Around half a billion WRA are anemic globally, with anaemia being higher in low and middle-income countries (LMICs) like India [14, 25]. The most recent National Family Health Survey (NFHS) reveals a significant burden of anaemia, impacting 57% of WRA in India, while in the Aspirational Districts, the prevalence of anaemia among WRA is even higher at 61% [26]. Earlier research conducted in India identified that low socioeconomic status, limited educational attainment, increased childbearing, residing in rural areas, women belongs to lower social groups, and inadequate dietary intake were associated with anaemia among WRA [22, 27–31]. Furthermore, a study on urban India found that anaemia was associated with low serum ferritin levels indicating iron deficiency [32, 33]. It is noteworthy that these studies are primarily focused on broader population trends within the country. The significance of studying anaemia in Aspirational Districts is underscored by its correlation with low economic status, a prevalent characteristic among women in Aspirational Districts [34–36].

The “Aspirational Districts” program was launched by the government of India in 2018. These districts were characterized by poor development indicators, high rates of poverty, and limited access to healthcare facilities, making them vulnerable to health and nutrition-related issues like anaemia [34, 37]. These districts were selected based on their poor performance across various development indicators, including health, education, nutrition, and basic infrastructure [37, 38]. Earlier research conducted in the Aspirational Districts predominantly focused on socioeconomic status, maternal and child health and the effectiveness of education, with no research specifically addressing anaemia which is a critical health issue prevalent among WRA [39–43]. By examining the prevalence of anaemia in Aspirational Districts, the research aims to inform targeted interventions aimed at reducing anaemia rates and improving the well-being of WRA in these districts.

Our study has the following objectives. First, our study measures the prevalence of anaemia among WRA in Aspirational Districts in NFHS-4 and NFHS-5. Second, the study aims to examine the factors associated with anaemia among WRA in Aspirational Districts. The study results may aid policymakers, public health researchers, and health professionals in better comprehending the shifting nature of anaemia across Aspirational Districts.

## Methods

### Data source

The study used the data from NFHS-4 and NFHS-5 conducted during 2015-16 and 2019-21, respectively. The NFHS aims to collect data on a diverse array of subjects, such as reproductive healthcare, fertility, infant and childhood mortality, HIVand acquired immune deficiency syndrome (AIDS), contraceptive awareness, family planning, maternal and infant nutrition, maternal and infant empowerment and more. Ministry of Health and Family Welfare, Government of India (MOHFW) conducted the study managed by the International Institute of Population Sciences (IIPS), Mumbai. Using a two-stage stratified random sampling method in each phase, NFHS-4 (2015–16) conducted interviews with 699,686 women aged 15–49 years across 572,000 households. In comparison, NFHS-5 interviewed 724,115 women aged 15–49 years from a total of 636,669 households with a response rate of 97% in both rounds [26, 44].

### Study sample

The “Aspirational Districts” program was launched by the government of India in 2018 [36]. The program was a part of India’s broader development agenda, which aims to promote inclusive and sustainable development across the country [37, 45]. These districts were selected based on their poor performance across various development indicators, including health, education, nutrition, and basic infrastructure [45]. Health was an important component of this program, accounting for almost 30%, with a focus on indicators like anaemia among women. A total of 112 districts from 27 states were identified as “Aspirational Districts”. Among these districts, there were 19 from Jharkhand, 13 from Bihar, ten each from Chhattisgarh and Odisha, eight each in Uttar Pradesh and Madhya Pradesh, seven in Assam. Rajasthan and Maharashtra have five and four districts, respectively, while Andhra Pradesh and Telangana each have three. Additionally, Uttarakhand, Tamil Nadu, Punjab, Jammu and Kashmir, Gujarat, and Karnataka have two districts. On the other hand, Arunachal Pradesh, Haryana, Himachal Pradesh, Manipur, Meghalaya, Mizoram, Nagaland, Sikkim, and Tripura each contribute one district to the program [34, 38, 45, 46].

For our study, we opted to include Visakhapatnam and Vizianagaram districts from Andhra Pradesh, even though Alluri Sitharamaraju and Parvathipuram Manyam districts are part of the Aspirational Districts program. This decision was based on the fact that the NFHS-4 and NFHS-5 surveys, conducted in 2015-16 and 2019-21, respectively, covered Visakhapatnam and Vizianagaram as survey districts. It’s worth noting that Alluri Sitharamaraju and Parvathipuram Manyam districts were formed in 2022 from Visakhapatnam and Vizianagaram, respectively. This choice was made to maintain consistency with the time frame of the survey data. The detailed list of Aspirational Districts used for this study is given in Supplementary Table 2.

**Fig. 1:**
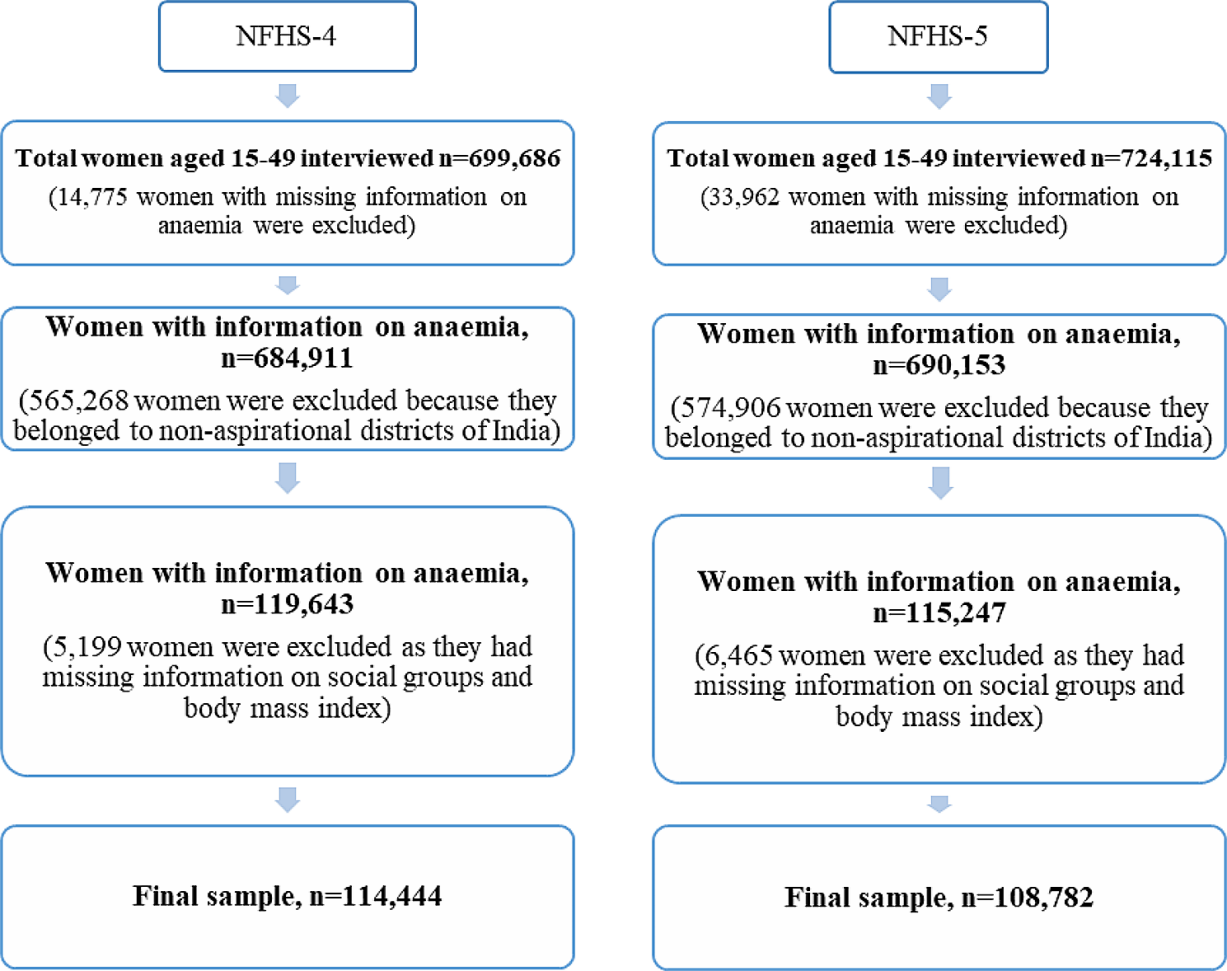
Process of sample selection for the study from NFHS-4 and NFHS-5

For the present study, data was extracted from two consecutive rounds of NFHS. A total of 114,444 and 108,782 women aged between 15 and 49 years were chosen from NFHS-4 and NFHS-5, respectively. The detailed sample selection process is given in Fig. 1.

### Hemoglobin testing

Health investigator took blood samples for anaemia testing from all women aged 15–49 who voluntarily consented to the testing. A drop of blood from a finger prick was used to collect blood samples in a microcuvette. In addition, on-site hemoglobin was tested using a battery-operated portable HemoCue Hb 201 + analyzer [26].

### Conceptual framework

**Fig. 2:**
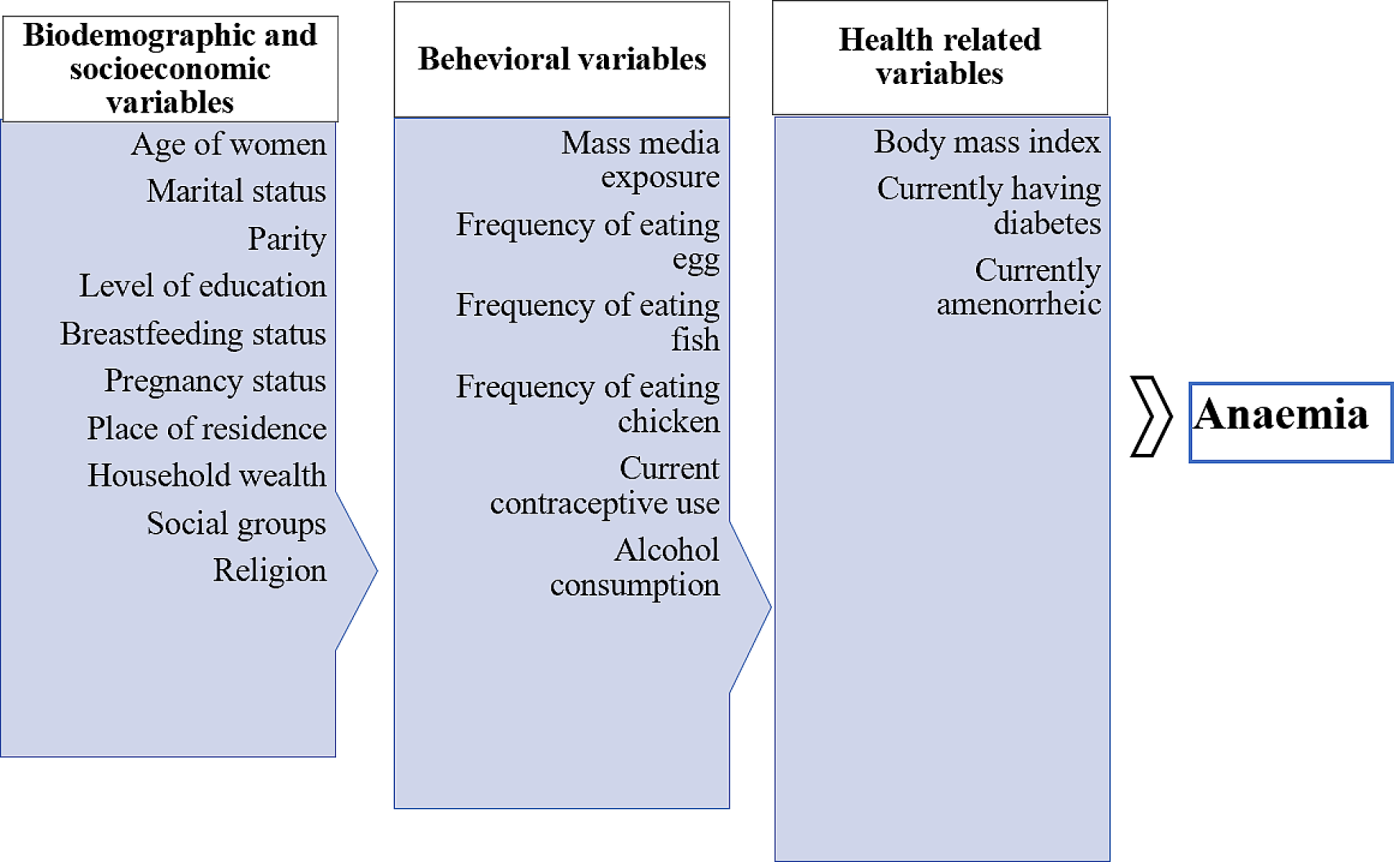
Conceptual framework showing determinants of anaemia

The framework illustrates various anaemia related variables which might affect the magnitude of anaemia in the Aspirational Districts of India. Three main domains of variables (biodemographic and socioeconomic, behavioural and health related variables) were selected for the study, which are described later in this section. The analysis for this study is based on this conceptual framework adapted from the existing literature on anaemia [47–53]. The conceptual framework is shown in Fig. 2.

### Dependent variable

The variable representing the level of anaemia in both the NFHS-4 and NFHS-5 datasets is an ordinal variable having four categories: no anaemia, mild anaemia, moderate anaemia, and severe anaemia. The level of anaemia is designated as mild if hemoglobin level is between 11.0 g/dl to 11.9 g/dl for non-pregnant women and 10.0 g/dl to 10.9 g/dl for pregnant women, moderate if hemoglobin level is between 8.0 g/dl to 10.9 g/dl for non-pregnant women and 7.0 g/dl to 9.9 g/dl for pregnant women and severe if hemoglobin level is within < 8.0 g/dl for non-pregnant women and < 7.0 g/dl for pregnant women [26]. The dependent variable in this study was whether reproductive women were anaemic or not anaemic. The dependent variable is coded as a dichotomous variable, with ‘1’ indicating “anaemic” for women with mild, moderate, or severe anaemic conditions, and ‘0’ indicating “not anaemic” for women having no anaemia.

### Independent variables

Based on prior research on anaemia, the independent variables were chosen for the study [32, 33, 47, 54–57]. These variables included age, marital status, parity, breastfeeding status, pregnancy status, level of education, social groups, religion, household wealth, type of residence, mass media exposure, frequency of eating eggs, frequency of eating fish, frequency of eating chicken, current contraceptive use, alcohol consumption, body mass index (BMI), currently having diabetes and currently amenorrhea. These variables were divided into three domains: (a) biodemographic and socioeconomic; (b) behavioural and (c) health related variables. Table 1 provides a detailed list of independent variables used in this study.

**Table 1:** Description of independent variables

Independent variables	Description (with codes)
**Biodemographic and socioeconomic variables**	
Age (in years)	Age of women was divided into four categories: ‘15–19 years’ (coded as ‘1’), ‘20–29 years’ (coded as’2’), ‘30–29 years’ (coded as ‘3’), ‘40–49 years’ (coded as ‘4’).
Marital status	Marital status has divided into three categories: ‘currently married’ (coded as ‘1’), ‘not married’ (coded as ‘2’), ‘formerly married’ (coded as ‘3’).
Parity	Parity was categorized into four: ‘no children’ (coded as ‘0’), ‘1–2 children’ (coded as ‘1’), ‘3–4 children’ (coded as ‘2’), ‘5 and above’ (coded as ‘3’).
Breastfeeding status	Breastfeeding status was categorized into two: ‘not breastfeeding’ (coded as ‘0’), ‘breastfeeding’ (coded as ‘1’).
Pregnancy status	Pregnancy status had two categories: ‘not pregnant’ (coded as ‘0’), ‘pregnant’ (coded as ‘1’).
Level of education	Education level of women was classified into four categories: ‘no education’ (coded as ‘0’), ‘primary’ (coded as ‘1’), ‘secondary’ (coded as ‘2’), ‘higher’ (coded as ‘3’).
Social groups	Social groups were divided into four categories: Scheduled Caste (SC) (coded as ‘1’), Scheduled Tribe (ST) (coded as ‘2’), Other Backward Classes (OBC) (coded as ‘3’), ‘Others’ (coded as ‘4’).
Religion	Religion was divided into four categories– ‘Hindu’ (coded as ‘1’), ‘Muslim’ (coded as ‘2’), ‘Christian’ (coded as ‘3’) ‘Others’ (coded as ‘4’).
Household wealth	The wealth index is a composite index of household amenities and assets; it indicates the socioeconomic condition; every household is given a score based on the number of consumer goods they own. A total of 33 assets and housing characteristics were taken into consideration to prepare a factor score using Principal Component Analysis. Thereafter this factor score was divided into five equal categories, − ‘poorest’ (coded as ‘1’); ‘poorer’ (coded as ‘2’); ‘middle’ (coded as ‘3’); ‘richer’ (coded as ‘4’); ‘richest’ (coded as ‘5’) each with 20% of the population.
Type of place of residence	Place of residence had two categories: ‘urban’ (coded as ‘0’), ‘rural’ (coded as ‘1’).
**Behavioral variables**	
Mass media exposure	Three questions were asked to women in NFHS-4 and NFHS-5. They are i) how often they read newspaper/magazines, ii) how often they watch television, and iii) how often they listen to radio. The responses are ‘almost every day’, ‘at least once a week’, ‘less than once a week’ and ‘not at all’. Based on these responses a composite index was computed and divided into four categories: ‘no’ (coded as ‘0’) if the respondent is not exposed to any mass media, ‘low’ (coded as ‘1’) if a respondent is exposed to any one type of mass media, ‘medium’ (coded as ‘2’) if the respondent is exposed to any two types of mass media, ‘high’ (coded as ‘3’) if the respondent is exposed to all three types of mass media.
Frequency of eating eggs	This variable was classified into three categories: ‘never’ (coded as ‘0’), ‘frequently’ (coded as ‘1’), ‘occasionally' (coded as ‘2’).
Frequency of eating fish	Frequency of eating fish was classified into three categories: ‘never’ (coded as ‘0’), ‘frequently’ (coded as ‘1’), ‘occasionally' (coded as ‘2’).
Frequency of eating chicken	This variable was classified into three categories: ‘never’ (coded as ‘0’), ‘frequently’ (coded as ‘1’), ‘occasionally' (coded as ‘2’).
Current contraceptive use	NFHS classified the contraceptive into 20 categories, all these categories. All these responses was recoded into two categories for this study: ‘no or traditional’ contraceptive use (coded as ‘0’), if a woman did not use anything or used periodic abstinence, withdrawal, other traditional method, or prolonged abstinence to delay or avoid getting pregnant; and ‘modern’ contraceptive use (coded as ‘1’) if a woman used pill, intrauterine device, injections, diaphragm, male condom, female sterilization, male sterilization, implants/norplant, lactational amenorrhea method, female condom, foam or jelly, emergency contraception, other modern method, standard days method, specific method 1, or specific method 2.
Alcohol consumption	Alcohol consumption was divided into two categories: ‘no’ (coded as ‘0’), ‘yes’ (coded as ‘1’)
**Health related variables**	
Body Mass Index	It is defined as the ratio between the weight of a women in kilogram divided by the squared height in meter. Body mass index was divided into four categories according to WHO cutoff; underweight (< 18.5 kg/m^2^) coded as ‘1’, normal (18.5 kg/m^2^–24.99 kg/m^2^) coded as ‘2’, overweight (25 kg/m^2^–32 kg/m^2^) coded as ‘3’ and obese (> 32 kg/m^2^) coded as ‘4’ [58].
Currently having diabetes	Self-reported diabetes had three categories: ‘no’ (coded as ‘0’) ‘yes’ (coded as ‘1’) ‘don’t know’ (coded as ‘8’).
Currently amenorrheic	Amenorrhea is the absence of menstruation, typically defined by the absence of one or more menstrual cycles. Amenorrhea had two categories: ‘no’ (coded as ‘0’), ‘yes’ (coded as ‘1’)
Year	NFHS-4, conducted during 2015-16 was coded as ‘0’ and NFHS-5, conducted during 2019-21 was coded as ‘1’.

### Statistical analysis

The study analyzed the prevalence of anaemia among WRA in Aspirational Districts of India by their background characteristics. We additionally evaluated the temporal change in the prevalence of anaemia for each Aspirational District during the study period. This analysis allowed us to identify variations in anaemia prevalence at a more detailed level. The chi-square test was used to analyze the statistical significance of the association between outcome variable and each independent variable [59]. Furthermore, multivariable binary logistic regression was used to examine the association between dependent variable and independent variables [60]. The data from NFHS-4 and NFHS-5 were pooled for this analysis. By combining these datasets, we were also able to examine the independent effect of the survey year on the likelihood of anaemia [61].

Three models were constructed and a block-wise forward selection method was used to eliminate any variables that were statistically insignificant (*p* > 0.05). Variables were introduced in blocks, and only those with a *p* < 0.05 were included in the subsequent models. Model 1 included biodemographic and socioeconomic variables, including age, marital status, parity, breastfeeding status, pregnancy status, level of education, social groups, religion, household wealth, and type of place of residence. Model 2 included statistically significant variables from model 1 and behavioral variables including mass media exposure, frequency of eating egg, frequency of eating fish, frequency of eating chicken, current contraceptive use, alcohol consumption, and model 3 contained significant variables from model 2 and health-related variables including BMI, currently having diabetes, currently amenorrheic and year. Adjusted odds ratios (AOR), *p*-values (< 0.05), and 95% confidence intervals (CIs) were used to show the results of logistic regression models. In addition, variance inflation factors (VIFs) were calculated to determine the degree of multicollinearity [62] (see additional Table 1). The mean VIF value was under the threshold value of five in all the models, which indicated that multicollinearity was not a problem for the models.

Furthermore, we applied Nagelkerke R square and Hosmer & Lemeshow tests to assess the goodness-of-fit of the models. Firstly, we conducted Nagelkerke R square, this helped us see how likely our model was to produce the observed data, and a lower log likelihood meant a better fit [63]. Additionally, we conducted the Hosmer-Lemeshow goodness-of-fit test. The Hosmer-Lemeshow test helped us assess how well our model’s predictions matched the actual outcomes, and if the result were not significant, it suggested a good fit [64]. In addition, we examined Akaike information criterion (AIC), Bayesian information criterion (BIC) and log likelihood. Higher log likelihood, and low AIC and BIC values indicated a better fit. [65].

Stata 16 was used for statistical analysis, and the *‘Svyset’* command was utilized to adjust for the complex survey design (sampling weights) of the NFHS-4 and NFHS-5 [66].

## Results

### Respondents’ characteristics

Table 2 presents the socio-demographic profile of WRA of Aspirational Districts. In both rounds of the NFHS, it was found that more than one-third of women were aged between 20 and 29, and around three-fourths of women were currently married. A minuscule percentage of women were pregnant and over 10% of women had five or more children. Furthermore, more than four-fifths women were identified as Hindu, and nearly half of the women belonged to OBC. Over one-third of women had not received any formal education and belonged to the lowest socioeconomic class. Contraceptive practices revealed that over 60% of women either used traditional methods or did not use any family planning method. In addition, across both rounds, over one-third of women reported that they occasionally ate egg, fish, and chicken. A small proportion of women in both rounds suffered from diabetes and amenorrhea.

**Table 2:** Background characteristics of WRA (15–49 years) in Aspirational Districts of India, NFHS-4 and NFHS-5.

	NFHS-4 (2015-16)		NFHS-5 (2019-21)	
Background characteristics	Frequency (*N* = 114,444)	%	Frequency (*N* = 108,782)	%
**Biodemographic and socioeconomic variables**				
**Age (in years)**				
15–19	21,856	19.1	20,459	18.8
20–29	39,256	34.3	36,834	33.9
30–39	29,767	26.0	28,609	26.3
40–49	23,565	20.6	22,881	21.0
**Marital status**				
Currently married	85,214	74.5	79,528	73.1
Not married	24,554	21.5	25,120	23.1
Formerly married	4,676	4.1	4,134	3.8
**Parity**				
No children	33,869	29.6	32,884	30.2
1–2 children	37,749	32.9	37,253	34.3
3–4 children	29,887	26.1	28,895	26.6
5 and above	12,939	11.3	9,751	8.9
**Breastfeeding status**				
Not breastfeeding	91,317	79.8	88,554	81.4
Breastfeeding	23,127	20.2	20,228	18.6
**Pregnancy status**				
Not pregnant	108,276	94.6	103,659	95.3
Pregnant	6,168	5.4	5,123	4.7
**Level of education**				
No education	46,167	40.3	36,918	33.9
Primary	14,486	12.7	13,248	12.2
Secondary	45,474	39.7	48,087	44.2
Higher	8,317	7.3	10,530	9.7
**Social groups**				
SC	20,309	17.8	21,648	19.9
ST	20,834	18.2	20,352	18.7
OBC	55,227	48.3	50,405	46.3
Others	18,074	15.8	16,377	15.1
**Religion**				
Hindu	93,563	81.8	90,425	83.1
Muslim	14,612	12.8	12,666	11.6
Christian	2,619	2.3	2,554	2.4
Others	3,650	3.2	3,136	2.9
**Household wealth**				
Poorest	40,886	35.7	37,876	34.8
Poorer	27,090	23.7	26,730	24.6
Middle	20,041	17.5	19,557	17.9
Richer	15,233	13.3	15,060	13.8
Richest	11,194	9.8	9,559	8.8
**Type of place of residence**				
Urban	22,226	19.4	20,344	18.7
Rural	92,218	80.6	88,438	81.3
**Behavioral variables**				
**Mass media exposure**				
No	38,302	33.5	38,510	35.4
Low	46,040	40.2	59,664	54.9
Medium	26,832	23.5	10,609	9.8
High	3,270	2.9	0	0.00
**Frequency of eating egg**				
Never	27,410	23.9	24,156	22.2
Frequently	41,795	36.5	47,286	43.5
Occasionally	45,239	39.5	37,340	34.3
**Frequency of eating fish**				
Never	29,809	26.1	26,922	24.8
Frequently	33,777	29.5	38,277	35.2
Occasionally	50,858	44.4	43,583	40.1
**Frequency of eating chicken**				
Never	27,975	24.4	26,367	24.2
Frequently	34,130	29.8	38,594	35.5
Occasionally	52,339	45.7	43,820	40.3
**Current contraceptive use**				
No use or traditional method	78,293	68.4	64,653	59.4
Modern	36,151	31.6	44,127	40.6
**Alcohol consumption**				
No	111,887	97.8	107,435	98.8
Yes	2,557	2.2	1,347	1.2
**Health related variables**				
**Body Mass Index**				
Underweight	32,911	28.8	25,695	23.6
Normal weight	66,478	58.1	65,774	60.5
Overweight	11,691	10.2	13,389	12.3
Obese	3,364	2.9	3,925	3.6
**Currently having diabetes**				
No	111,911	97.8	106,695	98.1
Yes	1,254	1.1	1,552	1.4
Don’t know	1,278	1.1	534	0.5
**Currently amenorrheic**				
No	106,769	93.3	103,386	95.0
Yes	7,675	6.7	5,396	4.9

Note: N = Number of women; SC: Scheduled Caste; ST: Scheduled Tribe; OBC: Other Backward Classes; all percentages are weighted

### Prevalence of anaemia among WRA by background characteristics

In NFHS-4, approximately 59% of WRA in Aspirational Districts were reported as anaemic. However, this percentage increased to 61% in NFHS-5. Table 3 presents the prevalence of anaemia among WRA from 2015 to 2021 in Aspirational Districts of India by background characteristics. In both rounds of the NFHS, the rates of anaemia remained nearly constant across all age groups, hovering around 60%. Furthermore, women with three to four children consistently exhibited the highest anaemia prevalence. However, the rate of anaemia increase was more pronounced among women with no children. Additionally, in both rounds of survey, the prevalence of anaemia was higher among women with no education, whereas those with higher education consistently had the lowest prevalence. In terms of household wealth, it was found that anaemia was higher among poorest category of women and lowest among the richest category.

**Table 3:** Prevalence of anaemia among WRA (15–49 years) by background characteristics in Aspirational Districts of India, NFHS-4 and NFHS-5.

	NFHS 4 (2015-16)		NFHS 5 (2019-21)	
Background characteristics	Prevalence of anaemia (Weighted %)	95% CI [Lower, Upper]	Prevalence of anaemia (Weighted %)	95% CI [Lower, Upper]
**Biodemographic and socioeconomic variables**				
**Age (in years)**	**χ**^**2**^ **= 14.61,** ***p*-value: 0.0273**		**χ**^**2**^ **= 28.06,** ***p*-value: 0.0004**	
15–19	58.7	[57.7,59.6]	62.4	[61.4,63.3]
20–29	59.3	[58.6,60.1]	61.3	[60.6,62.1]
30–39	57.9	[57.1,58.7]	60.2	[59.4,61.0]
40–49	58.6	[57.7,59.5]	60.5	[59.5,61.4]
**Marital status**	**χ**^**2**^ **= 33.69,** ***p***-value: **<0.001**		**χ**^**2**^ **= 15.30,** ***p***-value: **0.0078**	
Currently married	58.9	[58.3,59.4]	60.8	[60.2,61.4]
Not married	57.5	[56.6,58.4]	61.4	[60.5,62.3]
Formerly married	61.8	[59.9,63.7]	63.6	[61.8,65.4]
**Parity**	**χ**^**2**^ **= 46.63,** ***p*-value: <0.001**		**χ**^**2**^ **= 13.91, p-value: 0.0393**	
No child	57.2	[56.4,58.0]	60.5	[59.7,61.3]
1–2 children	58.9	[58.2,59.8]	60.9	[60.1,61.6]
3–4 children	59.6	[58.8,60.4]	61.9	[60.1,62.7]
5 and above	59.6	[58.4,60.7]	61.2	[59.9,62.5]
**Breastfeeding status**	**χ**^**2**^ **= 266.66,** ***p*-value: <0.001**		**χ**^**2**^ **= 167.05,** ***p*-value: <0.001**	
Not breastfeeding	57.5	[56.9,58.1]	60.1	[59.5,60.7]
Breastfeeding	63.4	[62.5,64.3]	65.1	[64.1,65.9]
**Pregnancy status**	**χ**^**2**^ **= 6.54, p-value: 0.0475**		**χ**^**2**^ **= 22.66, p-value: 0.0003**	
Not pregnant	58.8	[58.2,59.3]	61.2	[60.6,61.8]
Pregnant	57.1	[55.5,58.7]	57.9	[56.1,59.7]
**Level of education**	**χ**^**2**^ **= 346.51, p-value: <0.001**		**χ**^**2**^ **= 119.79, p-value: <0.001**	
No education	61.9	[60.9,62.3]	62.6	[61.8,63.4]
Primary	58.6	[57.5,59.8]	60.9	[59.9,61.1]
Secondary	56.9	[56.1,57.6]	60.8	[60.1,61.5]
Higher	52.7	[51.0,54.3]	56.8	[55.4,58.1]
**Social groups**	**χ**^**2**^ **= 966.08, p-value: <0.001**		**χ**^**2**^ **= 703.49, p-value: <0.001**	
SC	59.7	[58.6,60.8]	61.3	[60.3,62.3]
ST	66.9	[65.9,67.9]	68.9	[67.9,69.9]
OBC	57.4	[56.6,58.1]	58.6	[57.8,59.3]
Others	51.9	[50.8,53.1]	58.5	[57.2,59.8]
**Religion**	**χ**^**2**^ **= 215.24, p-value: <0.001**		**χ**^**2**^ **= 118.05, p-value: <0.001**	
Hindu	59.2	[58.6,59.8]	61.4	[60.8,62.0]
Muslim	53.6	[52.3,54.9]	57.0	[55.5,58.5]
Christian	60.9	[58.3,63.4]	62.8	[60.3,65.3]
Others	64.2	[60.8,67.5]	65.1	[62.8,67.4]
**Household wealth**	**χ**^**2**^ **= 610.72, p-value: <0.001**		**χ**^**2**^ **= 556.82, p-value: <0.001**	
Poorest	62.9	[62.1,63.6]	64.7	[63.9,65.6]
Poorer	58.6	[57.6,59.5]	61.9	[61.0,62.7]
Middle	57.3	[56.2,58.3]	59.3	[58.2,60.4]
Richer	53.8	[52.6,55.1]	57.3	[56.1,58.4]
Richest	52.9	[51.2,54.6]	53.7	[52.1,55.3]
**Type of place of residence**	**χ**^**2**^ **= 120.49, p-value: <0.001**		**χ**^**2**^ **= 89.87, p-value: <0.001**	
Urban	55.4	[54.2,56.7]	58.1	[56.7,59.5]
Rural	59.5	[58.8,60.1]	61.7	[61.1,62.3]
**Behavioral variables**				
**Mass media exposure**	**χ**^**2**^ **= 314.73, p-value: <0.001**		**χ**^**2**^ **= 179.13, p-value: <0.001**	
No	61.6	[60.8,62.3]	63.7	[62.9,64.5]
Low	58.8	[58.1,59.5]	59.8	[58.2,60.5]
Medium	55.1	[54.1,56.2]	58.5	[57.1,59.1]
High	52.8	[50.6,55.1]	0.00	0.00
**Frequency of eating egg**	**χ**^**2**^ **= 159.04, p-value: <0.001**		**χ**^**2**^ **= 62.01, p-value: <0.001**	
Never	55.5	[54.6,56.5]	59.0	[58.1,60.0]
Frequently	59.2	[58.3,60.0]	61.2	[60.5,61.9]
Occasionally	60.2	[59.5,60.9]	62.2	[61.4,63.0]
**Frequency of eating fish**	**χ**^**2**^ **= 281.25, p-value: <0.001**		**χ**^**2**^ **= 112.13, p-value: <0.001**	
Never	54.8	[53.9,55.8]	58.3	[57.4,59.3]
Frequently	58.8	[57.9,59.8]	61.8	[60.9,62.6]
Occasionally	60.8	[60.1,61.5]	62.1	[61.3,62.9]
**Frequency of eating chicken**	**χ**^**2**^ **= 217.46, p-value: <0.001**		**χ**^**2**^ **= 78.49, p-value: <0.001**	
Never	54.9	[54.1,55.9]	58.9	[58.0,59.9]
Frequently	59.4	[58.4,60.3]	61.0	[60.2,61.9]
Occasionally	60.2	[59.5,60.9]	62.3	[61.6,63.1]
**Current contraceptive use**	**χ**^**2**^ **= 21.66, p-value: 0.0009**		**χ**^**2**^ **= 38.64, p-value: <0.001**	
No or traditional	59.1	[58.5,59.7]	61.8	[61.2,62.5]
Modern	57.7	[56.8,58.5]	59.9	[59.2,60.7]
**Alcohol consumption**	**χ**^**2**^ **= 56.01, p-value: <0.001**		**χ**^**2**^ **= 65.08, p-value: <0.001**	
No	58.5	[58.0,59.1]	60.9	[60.4,61.5]
Yes	65.9	[63.4,68.3]	71.7	[69.0,74.3]
**Health related variables**				
**Body Mass Index**	**χ**^**2**^ **= 621.14, p-value: <0.001**		**χ**^**2**^ **= 438.68, p-value: <0.001**	
Underweight	62.9	[61.1,63.6]	65.0	[64.1,65.9]
Normal weight	58.4	[57.7,59.0]	61.1	[60.5,61.7]
Overweight	50.7	[49.4,52.0]	54.9	[53.7,56.1]
Obese	51.3	[48.5,54.0]	55.4	[53.1,57.6]
**Currently having diabetes**	**χ**^**2**^ **= 25.63, p-value: 0.0009**		**χ**^**2**^ **= 10.29, p-value: 0.0493**	
No	58.8	[58.2,59.3]	61.1	[60.5,61.7]
Yes	51.7	[47.9,55.5]	57.2	[53.8,60.5]
Don’t know	58.6	[54.9,62.1]	59.8	[54.5,64.9]
**Currently amenorrheic**	**χ**^**2**^ **= 131.09, p-value: <0.001**		**χ**^**2**^ **= 95.91, p-value: <0.001**	
No	58.2	[57.7,58.8]	60.7	[60.2,61.3]
Yes	64.9	[63.5,66.3]	67.4	[65.8,68.9]
**Year**	58.7	[58.1,59.2]	61.1	[60.5,61.6]

Note: Chi-square test applied for each variable, CI: Confidence interval

The prevalence of anaemia also varied among different social groups. Specifically, the rates were relatively higher among ST women during both surveys. However, the rate of increase in anaemia prevalence was notable among women from other social groups, with a 6 percentage point (pp) rise. Furthermore, during both rounds, the prevalence of anaemia was also higher among women those who occasionally ate eggs, fish and chicken. Although, the rate of increase in anaemia prevalence was more pronounced among WRA those who never consumed any of abovementioned food, with a nearly 4 pp rise. Women utilizing modern contraceptive methods exhibited a lower prevalence of anaemia compared to those using traditional or no contraceptive methods. Additionally, in NFHS-5, over 70% of WRA who consumed alcohol were found to be anaemic. Across both survey rounds, underweight women consistently exhibited the highest prevalence of anaemia, whereas overweight women consistently had the lowest prevalence. Notably, the increase in anaemia prevalence was more notable among overweight women, showing an increase of over 4 pp between the two survey rounds. Furthermore, women with diabetes were less likely to experience anaemia. However, in the case of amenorrhea, the prevalence of anaemia was highest among women with amenorrhea and lowest among those without amenorrhea in both rounds of NFHS.

### Distribution of anaemia among WRA in Aspirational Districts of India during NFHS-4 and NFHS-5

**Fig. 3:**
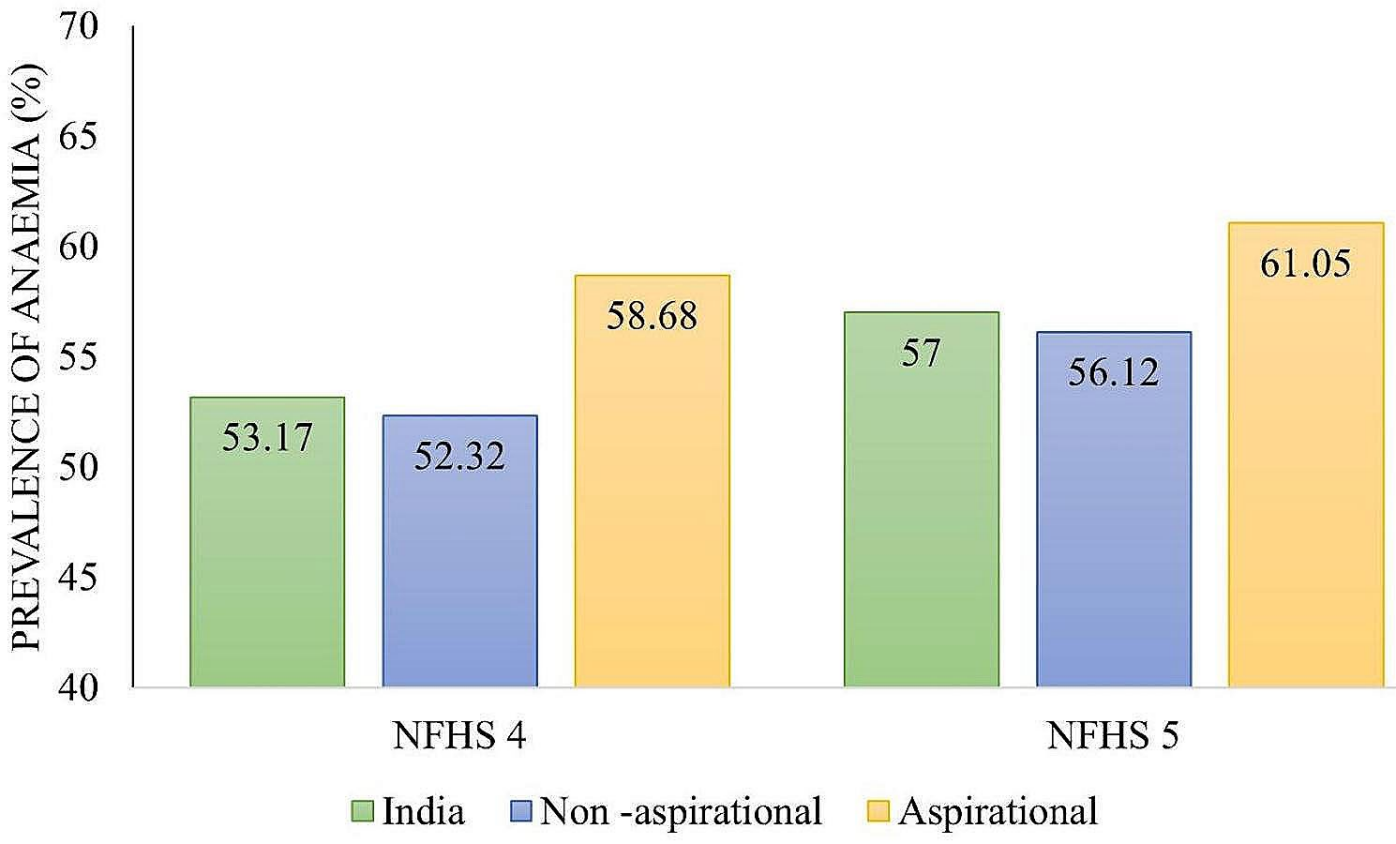
Prevalence of anaemia in Aspirational and non-aspirational Districts in NFHS-4 and NFHS-5.

Figure 3 illustrates that in both rounds of the NFHS, the prevalence of anaemia among WRA in Aspirational Districts exceeded that of non-aspirational districts and the national average. Nationally, 53.2% and 57% of WRA in India were anemic in NFHS-4 and NFHS-5, respectively. In contrast, the prevalence in Aspirational Districts was 58.7% and 61.1% in NFHS-4 and NFHS-5, respectively.

Between 2015 and 2021, more than 60% of all Aspirational Districts (72 out of 112) observed an increase in anaemia prevalence. On the other hand, 40 Aspirational Districts demonstrated a decline in anaemia prevalence during this period. There has been a notable increase in the number of Aspirational Districts where the prevalence of anaemia was 75% or higher. For instance, in NFHS-4, only two Aspirational Districts witnessed anaemia rates above 75%, but this number grew to nine in NFHS-5. Likewise, the number of Aspirational Districts with a prevalence of anaemia at 70% or higher increased from 13 in NFHS-4 to 21 in NFHS-5. In addition, 11 Aspirational Districts saw a rise of more than 20 pp, while 18 districts experienced a rise by at least ten pp. Additionally, there were Aspirational Districts where anaemia declined during the period from 2015 to 2021, with 8 Aspirational Districts experiencing a reduction by ten or more pp (see additional Table 2).

Aspirational Districts from Chhattisgarh witnessed a significant increase in anaemia, with 9 out of 10 districts experiencing a rise. Similarly, Bihar faced notable concerns, as 10 out of its 13 Aspirational Districts reported an increase in anaemia. Additionally, 6 out of 10 Aspirational Districts of Odisha observed an increase in anaemia. In contrast, states like Jharkhand, where 12 out of 19 Aspirational Districts experienced a decline in anaemia. Similarly, 7 out of 8 Aspirational Districts of Uttar Pradesh witnessed a decline in anaemia among WRA from 2015 to 2021 (see additional Table 2).

Additional Table 2 also shows, during both rounds, Chandel district of Manipur consistently experienced the highest anaemia among all Aspirational Districts, with 78% and 81%, respectively. On the other side, Simdega of Jharkhand in NFHS-4 and Udalguri of Assam in NFHS-5, witnessed the lowest anaemia prevalence, with at 23% and 27%, respectively. In addition, during both rounds, Barpeta followed by Udalgiri of Assam witnessed the maximum increase in anaemia with 29.4% pp and 26.7% pp, respectively. Conversely, Chitrakoot of Uttar Pradesh and Kalahandi of Odisha observed the maximum decline in anaemia with − 19.8% pp and − 21.1% pp, respectively.

### Determinants of anaemia among WRA in Aspirational Districts of India

Table 4 provides the AORs of anaemia among WRA in Aspirational Districts of India. We have found that, model 3 has lowest value of AIC and BIC and highest value of log likelihood and Hosmer-Lemeshow test, which indicate that model 3 is the best fit model among all the models.

**Table 4:** Adjusted odds ratios (with 95% CI) for anaemia among WRA (15–49 years) in Aspirational Districts of India, NFHS-4 and NFHS-5.

	Model 1				Model 2				Model 3			
		95% CI				95% CI				95% CI		
Background characteristics	AOR	Lower	Upper	*P*-value	AOR	Lower	Upper	*P*-value	AOR	Lower	Upper	*P*-value
**Biodemographic and socioeconomic variables** **Age (in years)**												
15–19®												
20–29	0.94	0.91	0.97	< 0.001	0.93	0.90	0.96	< 0.001	0.95	0.92	0.98	0.003
30–39	0.89	0.85	0.92	< 0.001	0.89	0.85	0.92	< 0.001	0.93	0.89	0.97	< 0.001
40–49	0.93	0.89	0.97	0.001	0.93	0.89	0.97	0.001	0.98	0.94	1.03	0.402
**Marital status**												
Not married®												
Currently married	0.93	0.89	0.96	< 0.001	0.93	0.90	0.97	< 0.001	0.96	0.92	1.00	0.055
Formerly married	1.06	1.00	1.12	0.048	1.05	0.99	1.11	0.117	1.07	1.01	1.14	0.016
**Parity**												
No child®												
1–2 children	1.06	1.02	1.10	0.002	1.08	1.04	1.13	< 0.001	1.09	1.05	1.13	< 0.001
3–4 children	1.08	1.04	1.13	< 0.001	1.12	1.07	1.17	< 0.001	1.13	1.08	1.17	< 0.001
5 and above	1.04	0.99	1.09	0.093	1.07	1.02	1.13	0.006	1.08	1.03	1.14	0.002
**Breastfeeding status**												
Not breastfeeding®												
Breastfeeding	1.24	1.21	1.28	< 0.001	1.22	1.19	1.25	< 0.001	1.17	1.13	1.20	< 0.001
**Pregnancy status**												
Not pregnant®												
Pregnant	0.88	0.85	0.92	< 0.001	0.86	0.82	0.90	< 0.001	0.89	0.85	0.93	< 0.001
**Level of education**												
No education®												
Primary	0.91	0.88	0.93	< 0.001	0.92	0.89	0.95	< 0.001	0.92	0.90	0.95	< 0.001
Secondary	0.92	0.90	0.94	< 0.001	0.94	0.92	0.96	< 0.001	0.93	0.90	0.95	< 0.001
Higher	0.87	0.84	0.91	< 0.001	0.90	0.87	0.94	< 0.001	0.87	0.84	0.91	< 0.001
**Social groups**												
Others®												
SC	1.14	1.11	1.18	< 0.001	1.12	1.09	1.16	< 0.001	1.10	1.07	1.14	< 0.001
ST	1.47	1.42	1.52	< 0.001	1.42	1.38	1.47	< 0.001	1.39	1.35	1.44	< 0.001
OBC	1.05	1.03	1.08	< 0.001	1.04	1.01	1.07	0.004	1.03	1.00	1.05	0.049
**Religion**												
Hindu®												
Muslim	0.92	0.89	0.94	< 0.001	0.87	0.84	0.89	< 0.001	0.88	0.85	0.90	< 0.001
Christian	0.59	0.57	0.61	< 0.001	0.57	0.55	0.60	< 0.001	0.59	0.57	0.62	< 0.001
Others	0.98	0.94	1.03	0.441	0.99	0.95	1.03	0.593	1.01	0.97	1.06	0.550
**Household wealth**												
Richest®												
Poorest	1.48	1.42	1.54	< 0.001	1.35	1.29	1.41	< 0.001	1.27	1.22	1.33	< 0.001
Poorer	1.28	1.23	1.33	< 0.001	1.20	1.15	1.25	< 0.001	1.15	1.11	1.20	< 0.001
Middle	1.17	1.13	1.22	< 0.001	1.12	1.07	1.16	< 0.001	1.08	1.04	1.13	< 0.001
Richer	1.08	1.04	1.13	< 0.001	1.05	1.01	1.09	0.008	1.03	1.00	1.07	0.087
**Type of place of residence**												
Urban®												
Rural	0.97	0.94	0.99	0.011	0.97	0.95	1.00	0.021	0.95	0.93	0.98	< 0.001
**Behavioral variables**												
**Mass media exposure**												
No®												
Low					0.95	0.93	0.97	< 0.001	0.96	0.94	0.99	0.001
Medium					0.88	0.85	0.91	< 0.001	0.95	0.92	0.98	0.001
High					0.80	0.74	0.86	< 0.001	0.90	0.83	0.97	0.008
**Frequency of eating egg**												
Never®												
Frequently					1.04	1.00	1.08	0.080				
Occasionally					1.00	0.97	1.04	0.848				
**Frequency of eating fish**												
Never®												
Frequently					1.12	1.07	1.17	< 0.001	1.13	1.11	1.16	< 0.001
Occasionally					1.14	1.10	1.19	< 0.001	1.14	1.12	1.17	< 0.001
**Frequency of eating chicken**												
Never®												
Frequently					0.98	0.94	1.03	0.507				
Occasionally					0.98	0.94	1.03	0.458				
**Current contraceptive use**												
No or traditional®												
Modern					0.93	0.91	0.95	< 0.001	0.92	0.90	0.94	< 0.001
**Alcohol consumption**												
No®												
Yes					1.04	0.99	1.10	0.147				
**Health related variables**												
**Body Mass Index**												
Normal weight®												
Underweight									1.20	1.18	1.23	< 0.001
Overweight									0.81	0.79	0.84	< 0.001
Obese									0.84	0.80	0.88	< 0.001
**Currently having diabetes**												
No®												
Yes									0.94	0.87	1.02	0.131
**Currently amenorrheic**												
No®												
Yes									1.13	1.09	1.18	< 0.001
**Year**												
NFHS 4®												
NFHS 5									1.19	1.17	1.22	< 0.001
**Tests for model fit**												
AIC	297463.05				297217.37				296238.72			
BIC	-3792.79				-3933.39				-4893.40			
Log likelihood	-148696.53				-148556.68				-148064.36			
Hosmer-Lemeshow chi^2^	39.11 (*p*-value: 0.042)				46.11 (*p*-value: 0.059)				41.59 (*p*-value: 0.093)			

Note: AIC: Akaike information criterion, BIC: Bayesian information criterion, AOR: Adjusted odds ratio, ®: Reference category

The final regression model (model 3) revealed that women in NFHS-5 had a 19% higher likelihood of having anaemia (AOR: 1.19, 95% CI: 1.17–1.21) compared to NFHS-4. Women with three to four children faced 13% higher odds of anaemia (AOR: 1.13, 95% CI: 1.08–1.18) compared to those without children. In addition, breastfeeding women had 17% higher (AOR: 1.17, 95% CI: 1.13–1.20) odds of being anaemic than non-breastfeeding women. Women with higher education had 13% lower odds of being anaemic (AOR: 0.87, 95% CI: 0.84–0.91), followed by those with primary education (AOR: 0.92, 95% CI: 0.90–0.95). Moreover, the odds of anaemia were higher among ST women (AOR: 1.39, 95% CI: 1.35–1.44) as compared to the women from other category. Christian women had a 40% lower likelihood of being anaemic (AOR: 0.60, 95% CI: 0.58–0.62) than Hindu women. Additionally, household wealth status showed a negative association with anaemia; women from the poorest quintile had 28% higher odds of anaemia (AOR: 1.28, 95% CI: 1.23–1.34) than the richest quintile. Furthermore, the odds of anaemia were 14% higher (AOR: 1.14, 95% CI: 1.12–1.17) among women who ate fish occasionally. Underweight women were 20% more likely to have anaemia (AOR: 1.20, 95% CI: 1.18–1.23) compared to those with normal weight. Additionally, women with amenorrhea had 13% higher odds of anaemia (AOR: 1.13, 95% CI: 1.08–1.17).

## Discussion

This study examined the prevalence of anaemia among WRA in Aspirational Districts of India between 2015 and 2021. During NFHS-4 and NFHS-5, the prevalence of anaemia among WRA within Aspirational Districts was higher than the national prevalence of anaemia. Within the Aspirational Districts, 72 districts experienced an increase, while 40 districts witnessed a decline in anaemia among WRA. Anaemia prevalence varied across Aspirational Districts, a majority of Aspirational Districts from Chhattisgarh and Bihar experienced a major rise in anaemia. While Aspirational Districts from Jharkhand and Uttar Pradesh observed a considerable decline in anaemia among WRA from 2015 to 2021. In terms of specific districts, Chandel of Manipur consistently faced the highest anaemia prevalence in both rounds of NFHS. In contrast, Simdega of Jharkhand and Udalguri of Assam witnessed the lowest anaemia prevalence in NFHS-4 and NFHS-5, respectively. During both rounds of survey, Barpeta and Udalgiri of Assam witnessed the highest increase in anaemia while, Chitrakoot of Uttar Pradesh and Kalahandi of Odisha witnessed highest decline in anaemia respectively.

After controlling for a number of factors, the regression results revealed that, women education, their wealth status, breastfeeding status, mass media exposure, social groups, underweight women, frequency of eating fish, and amenorrhea were associated with anaemia among WRA of Aspirational Districts of India.

This study found that women with higher education were less prone to be anaemic, this is supported by previous studies which identified women with higher education levels are less likely to develop anaemia than those with lower education levels [32, 47, 53, 67]. A possible reason could be, education provides knowledge and awareness about nutrition, health, and hygiene, which are essential for preventing anaemia [61, 68]. In addition, women with higher education levels are also more likely to have better access to healthcare, including prenatal care during pregnancy, which might help in managing anaemia [47].

Our study also revealed that poorest women had higher odds of anaemia compared to richest women. Poorest women face insufficient access to food resources, struggled to afford well-balanced meals, often reduced meal sizes, and occasionally skipped meals, all of which could contribute to anaemia [69–71]. Furthermore, knowledge of different vitamin and mineral- containing foods is generally limited among the lower wealth status women, which could be another factor contributing to anaemia [69, 72]. Furthermore, women with lower wealth status generally have limited access to healthcare services making diagnosing and treating anaemia more difficult [73, 74]. On the contrary, women belonging from the highest and richest households are associated with better nutritional status and were less vulnerable to get anaemia [69, 75, 76]. Additionally, lower wealth levels also lack resources for basic hygiene, such as clean water and sanitation facilities, which might increase the risk of infectious diseases that associate with anaemia [61].

Similar to many previous studies, this study also identified a positive association between anaemia and breastfeeding [25, 53, 61]. A study conducted in Pakistan revealed that inadequate consumption of enough iron-rich foods in their diet might cause insufficient milk supply, resulting in a shorter breastfeeding and the return of menstruation [25]. This, in turn, can contribute to iron deficiency and the beginning of anaemia [25, 77]. Though the relationship between breastfeeding and anaemia among WRA is little complex, which requires additional research for the better understanding.

The findings of the study indicated that increased mass media exposure was associated with a lower prevalence of anaemia. One possible explanation might be women with greater mass media exposure have greater access to information regarding the significance of nutrition and healthy behavior, such as consuming iron-rich foods or taking iron supplements [78]. Additionally, women with a significant exposure to mass media may be more likely to know about healthcare services, such as regular check-ups such as blood tests, which can aid in the early identification and treatment of anemia [79–81].

Our study also revealed that ST women were more likely to be anaemic than women from other social groups. Some previous studies have also revealed that anaemia is a significant health problem among tribal women in India [32, 48, 82, 83]. The higher anaemia prevalence in tribal women may have been a result of limited access to nutritious foods, including iron, folate, and vitamin B12 [84]. Moreover, dietary habits, poor sanitation practices, cultural norms like early marriage, insufficient maternal care, and taboos may collectively contribute to the prevalence of anemia among women of reproductive age in tribal communities [83, 85].

The relationship between fish consumption and anaemia is a bit complex. Some previous studies have indicated that anaemia tends to increase with fish consumption, a finding consistent with our own study [86]. However, there are some studies where it is found that anaemia does not increase with the consumption of fish [87, 88]. A possible explanation for these results may be that non-vegetarians have a higher animal protein intake, which can increase the body’s need for iron [89]. However, the underlying reasons for this complex relationship remain somewhat unclear and warrant further investigation.

Additionally, our study uncovered an association between underweight women and the prevalence of anemia. Previous studies have similarly shown that underweight women are associated with an elevated risk of anaemia [47, 67, 90, 91]. The potential cause for this could be insufficient intake of essential nutrients, such as iron and folate, and an inadequate diet, which are the predominant immediate causes of anaemia among WRA [67, 91]. Underweight individuals may also be more susceptible to chronic illnesses and inflammation which can interfere with the body’s inability to produce red blood cells, leading to anaemia [92, 93].

This study also identified an association between increased prevalence of anaemia and women who had experienced amenorrhea, consistent with earlier studies conducted in India [61]. This could be attributed to inadequate iron levels in the body, which typically result from improper and insufficient diet [25]. When the diet is inadequate, the body cannot produce an adequate amount of iron, leading to amenorrhea and subsequently resulting in anaemia among WRA [25, 77].

The Indian government has implemented a series of programs and policies aimed at preventing and eliminating anaemia among women. This began with the National Nutritional Anaemia Control Program (NNACP) in 1970, followed by the National Nutritional Policy in 1993. Additionally, Iron and Folic Acid Supplementation (WIFS) program, initiated in 2012, focused on providing iron and folic acid supplements to school-going and adolescent girls, aiming to prevent anaemia [94–96]. The National Iron + Initiative, launched in 2013, aimed to improve the quality and accessibility of iron and folic acid supplements in the public health system [97]. The Anaemia Mukt Bharat program (2018) aimed to increase awareness about anaemia, provide iron and folic acid supplements to vulnerable groups, and strengthen the health system for effective anaemia prevention and treatment [98]. Moreover, there are some states like Chhattisgarh and Uttar Pradesh where initiatives were adopted in order to eradicate NCDs including anaemia from Aspirational Districts [99, 100]. However, in addition to these initiatives, it is essential for more states to bring effective measures in Aspirational Districts to improve the condition of anaemia among WRA.

The major strength of this study is that this is the first national-level study to assess the prevalence of anaemia among WRA in Aspirational Districts using NFHS-4 and NFHS-5 datasets. Additionally, the study concentrated on assessing district-level anaemia prevalence and its temporal changes, aiming to inform the development of district-specific interventions to mitigate anaemia among women of reproductive age (WRA). Furthermore, the study studied the determinants of anaemia among WRA, which might help policy makers adopt specific measures to reduce anaemia among WRA in Aspirational Districts. One of the key limitations of this research arises from its cross-sectional survey design, which restricts causal inferences and instead permits only the establishment of associations between dependent and independent variables. An additional limitation is the exclusion of some key variables, such as folate, vitamin B12, vitamin A intake, access to healthcare services, and cultural beliefs and practices, which could influence anaemia prevalence but were not included due to their unavailability in the dataset. Furthermore, the reason of anaemia within WRA in Aspirational Districts is unknown because most Aspirational Districts research focuses on socioeconomic status, mother and child health, and education efficacy etc. Therefore, future research should incorporate more variables to provide a more comprehensive and accurate picture of the prevalence of anaemia among WRA of Aspirational Districts of India.

## Conclusion

Anaemia among WRA in Aspirational Districts was found higher than the national prevalence. Our study revealed that women with secondary education, ST women, poorest women, women who consume fish occasionally, underweight women and those with amenorrhea were associated with anaemia among WRA. The Government of India has taken several steps to reduce the prevalence of anaemia among women at national level, however there is a need to adopt some specific measures for Aspirational Districts too. More concerted efforts are needed to eradicate anaemia from Aspirational Districts. Focus towards healthy food consumption, better economic condition and higher education for women must be given importance. At the same time knowledge and awareness towards anaemia must be promoted among women. Implementing targeted public health programs in Aspirational Districts with higher prevalence of anaemia may reduce the issue at the grass-root level.

### Electronic supplementary material

Below is the link to the electronic supplementary material.

Supplementary Material 1

Supplementary Material 2

## Data Availability

All National Family Health Survey datasets used in this study are available at the official website of Demographic and Health Surveys (DHS): https://dhsprogram.com/data/availabledatasets.cfm. Additionally, this data can be obtained by registering as a DHS data user and requesting access for legitimate research purposes: https://dhsprogram.com/data/Access-Instructions.cfm.
