# Supplementary material for: Prevalence and determinants of anaemia among women of reproductive age in Aspirational Districts of India: an analysis of NFHS 4 and NFHS 5 data
Source: BMC Public Health. 2024 Feb 12;24:437. doi: 10.1186/s12889-024-17789-3 (PMC10860231; doi:10.1186/s12889-024-17789-3)
Supplement: Supplementary file 1 — Supplementary Material 1 [file 12889_2024_17789_MOESM1_ESM.docx]

**Additional Table 1: Variance inflation factors**

| **Background characteristics** | **VIF** | **1/VIF** |
| --- | --- | --- |
| **Biodemographic and socioeconomic factors** |  |  |
| **Age (in years)** |  |  |
| 15-19® |  |  |
| 20-29 | 3.26 | 0.31 |
| 30-39 | 4.13 | 0.24 |
| 40-49 | 4.16 | 0.24 |
| **Marital status** |  |  |
| Not married® |  |  |
| Currently married | 3.88 | 0.26 |
| Formerly married | 1.06 | 0.94 |
| **Parity** |  |  |
| No child® |  |  |
| 1-2 children | 4.11 | 0.24 |
| 3-4 children | 4.33 | 0.23 |
| 5 and above | 2.79 | 0.36 |
| **Pregnancy status** |  |  |
| Not pregnant® |  |  |
| Pregnant | 1.17 | 0.86 |
| **Level of education** |  |  |
| No education® |  |  |
| Primary | 1.27 | 0.78 |
| Secondary | 2.08 | 0.48 |
| Higher | 1.75 | 0.57 |
| **Social groups** |  |  |
| Others® |  |  |
| SC | 2.08 | 0.48 |
| ST | 2.10 | 0.48 |
| OBC | 1.82 | 0.55 |
| **Religion** |  |  |
| Hindu® |  |  |
| Muslim | 1.24 | 0.81 |
| Christian | 1.18 | 0.85 |
| Others | 1.07 | 0.93 |
| **Household wealth** |  |  |
| Richest® |  |  |
| Poorest | 1.44 | 0.69 |
| Poorer | 1.61 | 0.62 |
| Middle | 1.78 | 0.56 |
| Richer | 2.04 | 0.49 |
| **Type of place of residence** |  |  |
| Urban® |  |  |
| Rural | 1.35 | 0.74 |
| **Behavioral factors** |  |  |
| **Mass media exposure** |  |  |
| No® |  |  |
| Low | 1.66 | 0.60 |
| Medium | 1.92 | 0.52 |
| High | 1.12 | 0.89 |
| **Frequency of eating egg** |  |  |
| Never® |  |  |
| Frequently | 5.44 | 0.18 |
| Occasionally | 5.02 | 0.20 |
| **Frequency of eating fish** |  |  |
| Never® |  |  |
| Frequently | 4.95 | 0.20 |
| Occasionally | 5.06 | 0.20 |
| **Frequency of eating chicken** |  |  |
| Never® |  |  |
| Frequently | 6.54 | 0.15 |
| Occasionally | 6.65 | 0.15 |
| **Current contraceptive use** |  |  |
| No or traditional® |  |  |
| Modern | 1.48 | 0.68 |
| **Alcohol consumption** |  |  |
| No® |  |  |
| Yes | 1.08 | 0.93 |
| **Health related factors** |  |  |
| **Body Mass Index** |  |  |
| Normal weight® |  |  |
| Underweight | 1.38 | 0.72 |
| Overweight | 1.44 | 0.69 |
| Obese | 1.17 | 0.86 |
| **Currently having diabetes** |  |  |
| No® |  |  |
| Yes | 1.02 | 0.98 |
| **Currently amenorrheic** |  |  |
| No® |  |  |
| Yes | 1.15 | 0.87 |
| **Mean VIF** | 2.49 |  |

Note: ® Reference category.
