# Supplementary material for: Prevalence and determinants of anaemia among women of reproductive age in Aspirational Districts of India: an analysis of NFHS 4 and NFHS 5 data
Source: BMC Public Health. 2024 Feb 12;24:437. doi: 10.1186/s12889-024-17789-3 (PMC10860231; doi:10.1186/s12889-024-17789-3)
Supplement: Supplementary file 2 — Supplementary Material 2 [file 12889_2024_17789_MOESM2_ESM.docx]

**Additional Table 2: Distribution of anaemia in percentage and change in anaemia in percentage point among WRA (15-49 years) in aspirational districts of India during NFHS-4 (2015-16) and NFHS-5 (2019-21).**

| **Name of state** | **Name of district** | **NFHS-4 (%)** | **NFHS-5 (%)** | **Change (% points)** |
| --- | --- | --- | --- | --- |
| Assam | Barpeta | 35.45 | 64.83 | 29.38 |
| Assam | Udalguri | 54.76 | 81.49 | 26.73 |
| Odisha | Dhenkanal | 39.41 | 65.93 | 26.52 |
| Assam | Darrang | 45.07 | 70.39 | 25.32 |
| Rajasthan | Karauli | 38.07 | 61.48 | 23.41 |
| Uttar Pradesh | Fatehpur | 40.15 | 62.98 | 22.83 |
| Rajasthan | Dholpur | 46.52 | 68.48 | 21.96 |
| Chhattisgarh | Korba | 45.13 | 66.43 | 21.3 |
| Maharashtra | Washim | 35.46 | 56.35 | 20.89 |
| Gujarat | Narmada | 55.57 | 75.91 | 20.34 |
| Assam | Baksa | 53.4 | 73.56 | 20.16 |
| Gujarat | Dahod | 56.27 | 75.08 | 18.81 |
| Jammu & Kashmir | Baramulla | 55.57 | 72.47 | 16.9 |
| Madhya Pradesh | Chhatarpur | 48.1 | 63.5 | 15.4 |
| Assam | Goalpara | 49.72 | 64.88 | 15.16 |
| Chhattisgarh | Rajnandgaon | 43.68 | 58.27 | 14.59 |
| Maharashtra | Gadchiroli | 51.65 | 66.16 | 14.51 |
| Assam | Hailakandi | 47.16 | 61.36 | 14.2 |
| Odisha | Rayagada | 55.36 | 69.14 | 13.78 |
| Tripura | Dhalai | 56.43 | 69.99 | 13.56 |
| Chhattisgarh | Mahasamund | 49.48 | 63.03 | 13.55 |
| Bihar | Jamui | 61.87 | 75.16 | 13.29 |
| Chhattisgarh | Narayanpur | 58.93 | 71.97 | 13.04 |
| Maharashtra | Osmanabad | 36.35 | 49.14 | 12.79 |
| Chhattisgarh | Kondagaon | 67.59 | 79.74 | 12.15 |
| Bihar | Nawada | 58.84 | 70.41 | 11.57 |
| Odisha | Kandhamal | 52.71 | 64.23 | 11.52 |
| Rajasthan | Jaisalmer | 33.57 | 44.44 | 10.87 |
| Assam | Dhubri | 52.49 | 63.23 | 10.74 |
| Jharkhand | Dumka | 63.71 | 73.42 | 9.71 |
| Jharkhand | Latehar | 53.93 | 63.58 | 9.65 |
| Jharkhand | Sahibganj | 61.89 | 71.51 | 9.62 |
| Chhattisgarh | Bastar | 67.59 | 77.2 | 9.61 |
| Karnataka | Yadgir | 47.74 | 57.3 | 9.56 |
| Telangana | Warangal | 56.89 | 65.88 | 8.99 |
| Jharkhand | Pakur | 71.08 | 79.72 | 8.64 |
| Odisha | Gajapati | 58.48 | 66.12 | 7.64 |
| Jharkhand | Palamu | 53.62 | 61.02 | 7.4 |
| Mizoram | Mamit | 27.60 | 34.89 | 7.29 |
| Punjab | Moga | 47.46 | 54.38 | 6.92 |
| Bihar | Muzaffarpur | 52.43 | 58.88 | 6.45 |
| Madhya Pradesh | Khandwa | 58.54 | 64.83 | 6.29 |
| Bihar | Aurangabad | 54.29 | 60.45 | 6.16 |
| Jharkhand | Khunti | 64.11 | 70.02 | 5.91 |
| Sikkim | West District | 36.82 | 42.65 | 5.83 |
| Bihar | Katihar | 63.78 | 68.37 | 4.59 |
| Punjab | Firozpur | 57.01 | 61.45 | 4.44 |
| Rajasthan | Sirohi | 59.80 | 64.03 | 4.23 |
| Maharashtra | Nandurbar | 60.22 | 64.23 | 4.01 |
| Chhattisgarh | Sukma | 74.49 | 78.42 | 3.93 |
| Manipur | Chandel | 23.47 | 27.27 | 3.8 |
| Bihar | Begusarai | 59.25 | 62.89 | 3.64 |
| Madhya Pradesh | Guna | 46.20 | 49.78 | 3.58 |
| Telangana | Asifabad | 63.72 | 67.28 | 3.56 |
| Chhattisgarh | Bijapur | 68.73 | 72.14 | 3.41 |
| Arunachal Pradesh | Namsai | 55.28 | 58.56 | 3.28 |
| Tamil Nadu | Ramanathapuram | 50.33 | 53.55 | 3.22 |
| Jammu & Kashmir | Kupwara | 70.48 | 73.46 | 2.98 |
| Bihar | Sheikhpura | 66.76 | 69.52 | 2.76 |
| Madhya Pradesh | Damoh | 45.5 | 48.07 | 2.57 |
| Jharkhand | Garhwa | 60.13 | 62.69 | 2.56 |
| Bihar | Gaya | 61.78 | 64.33 | 2.55 |
| Bihar | Araria | 65.59 | 67.94 | 2.35 |
| Madhya Pradesh | Rajgarh | 50.27 | 52.33 | 2.06 |
| Bihar | Sitamarh | 59.8 | 61.68 | 1.88 |
| Nagaland | Kiphire | 29.62 | 31.39 | 1.77 |
| Karnataka | Raichur | 58.7 | 60.36 | 1.66 |
| Madhya Pradesh | Singrauli | 52.56 | 54.11 | 1.55 |
| Chhattisgarh | Dakshin Bastar | 74.49 | 76.01 | 1.52 |
| Tamil Nadu | Virudhunagar | 55.86 | 56.88 | 1.02 |
| Odisha | Malkangiri | 71.28 | 71.92 | 0.64 |
| Odisha | Nuapada | 64 | 64.02 | 0.02 |
| Jharkhand | Pashchimi Singhbhum | 72.79 | 72.57 | -0.22 |
| Bihar | Khagaria | 59.75 | 59.46 | -0.29 |
| Jharkhand | Chatra | 56.64 | 55.96 | -0.68 |
| Bihar | Banka | 66.99 | 65.86 | -1.13 |
| Uttarakhand | Udham Singh Nagar | 52.29 | 51.01 | -1.28 |
| Andhra Pradesh | Y.S.R. Kadapa | 57.69 | 56.13 | -1.56 |
| Odisha | Balangir | 61.05 | 59.35 | -1.7 |
| Uttar Pradesh | Balrampur | 55.8 | 53.7 | -2.1 |
| Jharkhand | Purbi Singhbhum | 66.59 | 64.37 | -2.22 |
| Chhattisgarh | Dantewada | 67.46 | 65.21 | -2.25 |
| Odisha | Nabarangpur | 71.52 | 69.25 | -2.27 |
| Telangana | Khammam | 71.15 | 68.71 | -2.44 |
| Bihar | Purnia | 68.8 | 66.03 | -2.77 |
| Jharkhand | Bokaro | 72.41 | 68.84 | -3.57 |
| Jharkhand | Giridih | 68.48 | 64.7 | -3.78 |
| Uttar Pradesh | Bahraich | 52.7 | 48.81 | -3.89 |
| Uttar Pradesh | Shrawasti | 48.74 | 44.41 | -4.33 |
| Jharkhand | Hazaribagh | 60.84 | 56.12 | -4.72 |
| Jharkhand | Lohardaga | 66.67 | 61.7 | -4.97 |
| Odisha | Koraput | 63.25 | 58.23 | -5.02 |
| Jharkhand | Godda | 71.57 | 66.27 | -5.3 |
| Jharkhand | Ramgarh | 67.56 | 62.26 | -5.3 |
| Kerala | Wayanad | 32.25 | 26.94 | -5.31 |
| Uttar Pradesh | Siddharthnagar | 56.56 | 51.17 | -5.39 |
| Jharkhand | Ranchi | 64.53 | 58.92 | -5.61 |
| Meghalaya | Ribhoi | 67.97 | 62.36 | -5.61 |
| Madhya Pradesh | Vidisha | 44.24 | 38.5 | -5.74 |
| Rajasthan | Baran | 66.28 | 60.07 | -6.21 |
| Madhya Pradesh | Barwani | 65.8 | 58.38 | -7.42 |
| Himachal Pradesh | Chamba | 51.23 | 43.71 | -7.52 |
| Jharkhand | Simdega | 78.21 | 70.64 | -7.57 |
| Andhra Pradesh | Visakhapatnam | 66.35 | 58.02 | -8.33 |
| Haryana | Mewat | 70.06 | 60.57 | -9.49 |
| Jharkhand | Gumla | 69.64 | 59.48 | -10.16 |
| Andhra Pradesh | Vizianagaram | 75.52 | 64.04 | -11.48 |
| Uttarakhand | Haridwar | 55.25 | 43.77 | -11.48 |
| Uttar Pradesh | Chandauli | 63.89 | 48.69 | -15.2 |
| Uttar Pradesh | Sonbhadra | 60.45 | 44.54 | -15.91 |
| Odisha | Kalahandi | 68.71 | 48.91 | -19.8 |
| Uttar Pradesh | Chitrakoot | 67.68 | 46.57 | -21.11 |
